# Research on the effectiveness of a intersection risk warning system based on driving simulation experiment

**DOI:** 10.1038/s41598-022-16297-x

**Published:** 2022-07-18

**Authors:** Yiwen Zhou, Fengxiang Guo, Wenchen Yang, Huasen Wan

**Affiliations:** 1 School of Transportation Engineering, Kunming University of Science and Technology, Kunming, 650500 Yunnan China; 2 National Engineering Laboratory for Surface Transportation Weather Impacts Prevention, Broadvision Engineering Consultants, Kunming, 650200 Yunnan China; 3 Yunnan Key Laboratory of Digital Communications, Yunnan, 650103 China

**Keywords:** Engineering, Civil engineering

## Abstract

In order to improve the traffic safety condition of intersections, a real-time traffic conflict risk warning system (RTCRWS) is proposed for uncontrolled intersections. To evaluate the effectiveness of this system, a driving simulation experiment was designed and conducted. In this study, a virtual experimental scene including static road, traffic environment and dynamic traffic flow was constructed, and 45 drivers were recruited to complete the driving simulation experiment at 13 intersections. Three different data analysis methods were employed: (1) descriptive analysis of driving behavior characteristics; (2) descriptive analysis of physiological and psychological reactions of drivers; (3) Technique for Order Preference by Similarity to an Ideal Solution (TOPSIS) of RTCRWS. The results show that RTCRWS can effectively control the vehicle speed and reduce the driver's tension. In addition, the following conclusions are also drawn: (1) The early warning signs with better warning effect among the two types signs of RTCRWS were compared; (2) Among the elderly and young and middle-aged drivers, RTCRWS has a better warning effect on the elderly drivers. (3) Among the male and female drivers, RTCRWS has a better warning effect on female drivers.

## Introduction

More than 1.35 million people die from road traffic accidents worldwide each year. Approximately 3,700 people die on roads every day^1^. Surprisingly, road traffic injuries are the leading cause of death among people aged 5–29 years^2^. Compared with roads in general areas, mountain roads have more frequent traffic accidents^3^. Among them, the risk of accidents at the intersections on mountain roads that are not signal controlled is greater, and the safety situation is severe^4^. To effectively improve the driving safety of this type of intersection, various intersection variable message signs (VMSs) and risk warning systems have been designed and utilized, and significant effects have been achieved.

The research on the VMS system includes information content, color scheme, appropriate amount of information, and the interaction of VMS. Lai^5^ showed that drivers respond to two-colour signs faster than one- or three-colour signs. Furthermore, drivers respond less to two-line messages than to single-line and three-line messages. Zhao et al.^6^ pointed out that, on foggy days, drivers prefer a single-line display of lane-changing advice information. In normal weather, people driving to work prefer text congestion reminders. In addition, Xu et al.^7^ showed that, as the number of roads displayed by a VMS increase, the speed, time, and understanding accuracy is reduced, and the maximum number of roads in VMS should be five. To solve the problem of VMS location selection, Li et al.^8^ proposed a model to optimise the design of VMSs. The study found that the travel time information provided by a VMS may reduce the performance of the overall transportation network.

A well-designed VMS can not only enable drivers to better locate exits and reduce the number of lane changes, but also improve traffic flow to within a certain range. Therefore, it is necessary to optimise VMSs. After comparing different setup schemes of leading signs, it is suggested that three advance guide signs should be set at 1, 0.5, and 0 km from the starting point of the conical deceleration lane. If the conditions are limited, at least two advance guide signs^9^ should be used. In addition, the factors affecting the readability of VMSs were studied, and the driver's behaviour was evaluated according to the duration of the two types of messages. The effect is better when a message is repeated twice in a short period of time^10^.

To evaluate whether a VMS can play the expected role of advance notice, flow control, deceleration, and lane change after setting, and to analyse the influence of the VMS on driving behaviour, the effectiveness of the VMS was evaluated. Guattari et al.^11^ indicated that when drivers do not understand road signs, the speed of an approaching VMS decreases by more than 5%. For specific driving behaviours (deceleration, lane change, etc.), the information released by 87.50% VMS failed to cause a speed change, while the information released by 71.85% VMS failed to cause the expected lane change, and heavy vehicle drivers and low-mileage drivers were more likely to follow the lane change information^12^. For different types of professional drivers (truck and taxi drivers), we analysed whether they would change lanes after being provided with real-time traffic information. The results indicated that VMS information is unrelated to the type of driver, but is related to individual differences of drivers. The driver's own factors^13^.

Presently, research on risk warning systems mainly includes the design of risk warning systems, display strategies, and the effectiveness of early warning information. The active warning system (RIAWS) at rural intersections was studied and designed, and the influence of sign content (80 km/h and deceleration) on drivers' instantaneous speed was compared and analysed. It was found that the "80 km/h" sign is more effective than the "Deceleration" sign^14^. Tu and Huang^15^ proposed a collision warning system for all road intersections. Based on the model of the minimum distance of the vehicle's future trajectory, a collision detection algorithm was designed, and a layered warning was given according to the situation. Mackiea et al.^16^ evaluated the relative effectiveness of the "70 km/h variable speed limit" sign and "deceleration" sign. The results revealed that most drivers have a good understanding of RIAWS, and variable speed limits will cause drivers to decelerate more significantly.

The intersection conflict warning system (ICWS) is designed to reduce the frequency of collisions by alerting drivers to conflicting vehicles on adjacent leads at unsignalized intersections. Some advanced intelligent intersection warning systems have been developed. Weidemann et al.^17^ proposed a new LED warning system that proactively detects all approaching vehicles and activates LED flashing warning signs for conflicting movements. The effectiveness of the system was further evaluated by intersection driver behavior analysis obtained from video data, as well as surveys of residents and frequent users of the intersection. A more advanced intelligent intersection warning system was developed that uses radar sensors to measure the position, speed, and acceleration of approaching vehicles. Then, the algorithm of the system will determine whether there is any potential conflict between the vehicles and activate the system to warn the driver^18^. After ICWS is set up, its effect and impact on driving should be further evaluated. A comprehensive evaluation of ICWS performance, cost and service life based on accident data revealed that collision accidents at two-lane and four-lane intersections were reduced by 5%, and the ICWS strategy was highly cost-effective even with conservative assumptions about the cost, service life, and the value of a statistical life^19^. The influence of ICWS on driver behavior is mainly reflected in the difference of driver behavior before and after the system is set up. Thapa et al.^20^ made a comparative analysis of the driving behavior of the approach with and without ICWS, and evaluated the spillover effect of ICWS on other adjacent controlled intersections without the application of ICWS. Data was collected using camera arrays over three different time horizons: before, one month after, and 12 months after system installation. Analysis of the data using the critical gap method indicated that ICWS improved driver gap acceptance only during the 12-month period after installation, and there were no "spillover effects" at adjacent control sites. When gap acceptability is further compared according to the type of stop, it is found that the critical gap choice increases for drivers who perform full and rolling stops.

In this study, a risk warning system for real-time perception of traffic conditions at intersections (RTCRWS) was proposed. A vehicle detector on the road detected a vehicle and transmitted this information to a roadside warning system. The roadside system then adjusted the display information of the warning signs in real time. To analyse the early warning effect of this type of risk warning system, questionnaire surveys and driving simulation experiments were conducted. The physiological and psychological data (e.g., HR and EDA) and driving data (e.g., speed, maximum brake pedal depth, and earliest braking time) were then used to study the effectiveness of the RTCRWS.

## Methodology

### Participants

The sample size is closely related to the reliability and validity of the experimental results. If the sample size is too small, the reliability of the results will be reduced, and if the sample size is too large, time and resources will be wasted. Therefore, the minimum sample size N required for the experiment were calculated based on the expected variance, target confidence and error margin, as shown in formula (1)^21^:

1 $$N = Z^{2} \sigma^{2} /E^{2}$$

where N is the sample size, σ is the standard deviation, Z is the standard normal distribution statistic, and E is the maximum error.

To confirm the minimum sample size required for the experiment, when the confidence level is 90%, Z is 1.25; the value of σ ranges from 0.25 to 0.50, and the value of this study is 0.45; E is 10%, and the calculated value of N is 31.

A total of 45 drivers participated in the study. Only 38 valid data were obtained due to discomfort and dizziness in the driving simulator. According to Article 2 of China's Law on the Protection of The Rights and Interests of the Elderly, 60 years of age is taken as the basis for the classification of the elderly. Elderly drivers and middle-aged and young drivers were selected according to 1:1 ratio (population ratio: elderly drivers: middle-aged and young drivers = 1.375:1, male:female = 1.375:1). Accordingly, three groups of participants were present: 16 elderly drivers (age 60–72, M = 64, SD = 3.22; 11 males and 5 females), 16 middle-aged drivers (age 35–60, M = 44, SD = 5.94; 7 males and 9 females), and 6 young drivers (age 21–35, M = 27, SD = 4.5; 6 males). All the participants had been driving for more than two years, with a driving mileage exceeding 20,000 km. They were in good physical and mental health, with no eye diseases, and could make independent decisions. And Informed consent was obtained from all subjects and/or their legal guardian(s).

### Apparatus

#### Driving simulator

**Figure 1:**
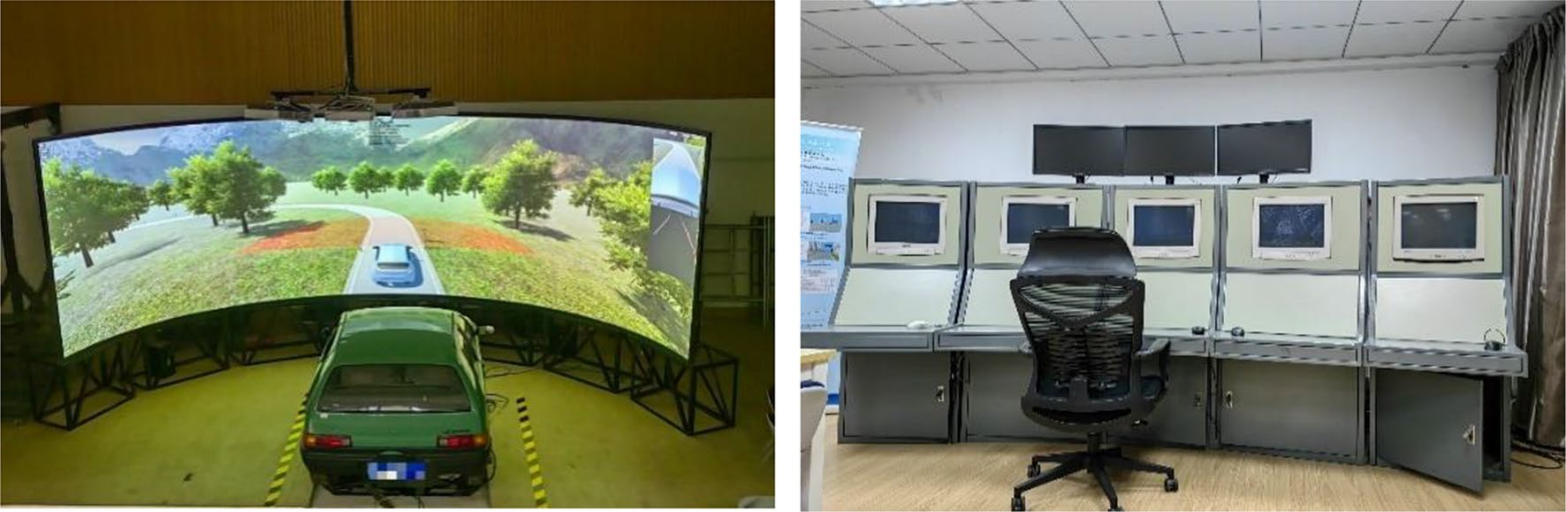
KMRTDS driving simulation system.

Driving simulations were performed using the KMRTDS driving simulation system developed by the Road Traffic Simulation Laboratory of the School of Traffic Engineering, Kunming University of Science and Technology, China, which has 6 degrees of freedom. The KMRTDS driving simulation system has a driving simulation control cabin, which can realise closed-loop driving simulation experiments of road traffic systems under laboratory conditions. The system is generally divided into two parts: a cockpit and a console. The appearance of the system is shown in the Fig. 1.

#### Physiological and psychological instrument

**Figure 2:**
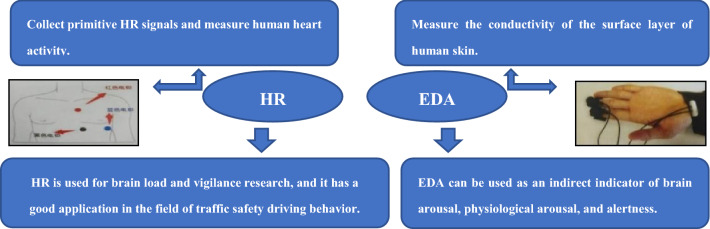
Physiological and psychological parameters.

In this experiment, ErgoLAB wearable wireless physiological instrument was used to collect the psychological and physiological data of the drivers. It is a wireless device that does not affect the real natural behavior of test drivers and is suitable for real-world field research. The physiological parameters measured during the experiment are shown in Fig. 2.

### Intersection experiment scenarios design

**Figure 3:**
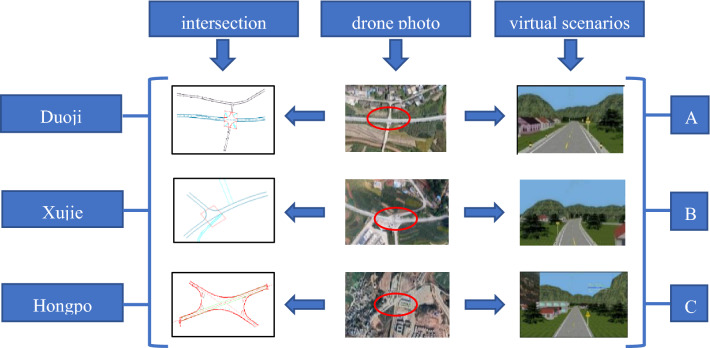
Plan and virtual scenarios of the three intersections.

**Figure 4:**
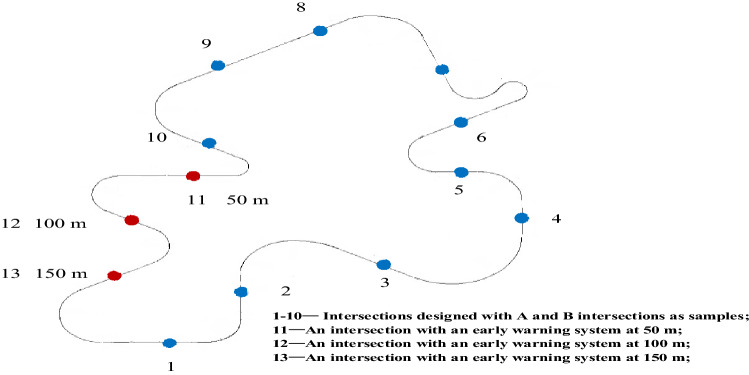
Road utilised in the simulation experiment.

**Figure 5:**
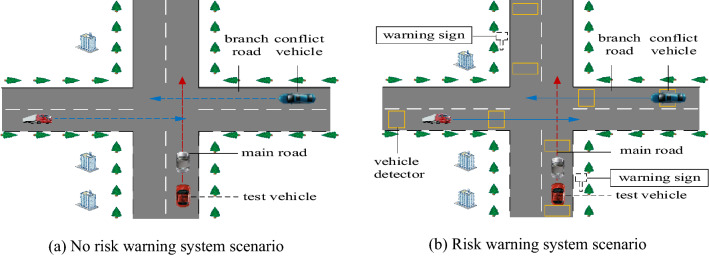
Intersection experiment scenarios.

This experiment was based on the road alignment data of rural roads in Yunnan Province, China, and three typical intersections were selected. According to statistics, from 2011 to 2017, more than 20 traffic accidents occurred at the intersections of Duoji, Xujie, and Hongpo due to poor visibility of the intersections, unreasonable intersection spatial layouts, and lack of signal control. The intersections of Duoji, Xujie, and Hongpo were restored in the VS-Design 3D scenario design software. The corresponding UAV photos, intersection plans, and virtual simulation scenarios are shown in Fig. 3. For convenience of description, the three intersections were defined as A, B, and C. To compare the effectiveness of the risk warning system, scenarios in which the intersections had warning systems and scenarios in which the intersections did not have warning systems were designed. The intersections of the experimental scenarios are shown in Fig. 5. To compare and analyse the warning effect of the risk warning system among elderly, middle-aged, young drivers, male and female drivers, different early warning signs were designed. Taking intersections A and B as the prototype intersections, a simulated experimental road with 13 intersections was designed (Fig. 4).

### Warning sign information display

The warning system proposed in this study is a new safety technology product developed under the new concept. In terms of size, it refers to GB 5768.2–2009, and human factors are considered in the design process. Real vehicle experiment and driver questionnaire are adopted to study the visual recognition of intelligent roadside warning facilities under different specifications and sizes, and the size of 1.0 m*1.0 m facilities is finally determined. Two types of warning signs suitable for the intersections on mountain roads that are not signal controlled were designed. The effectiveness evaluation results could inform the development of more effective warning information display strategies (Fig. 5).

**Figure 6:**
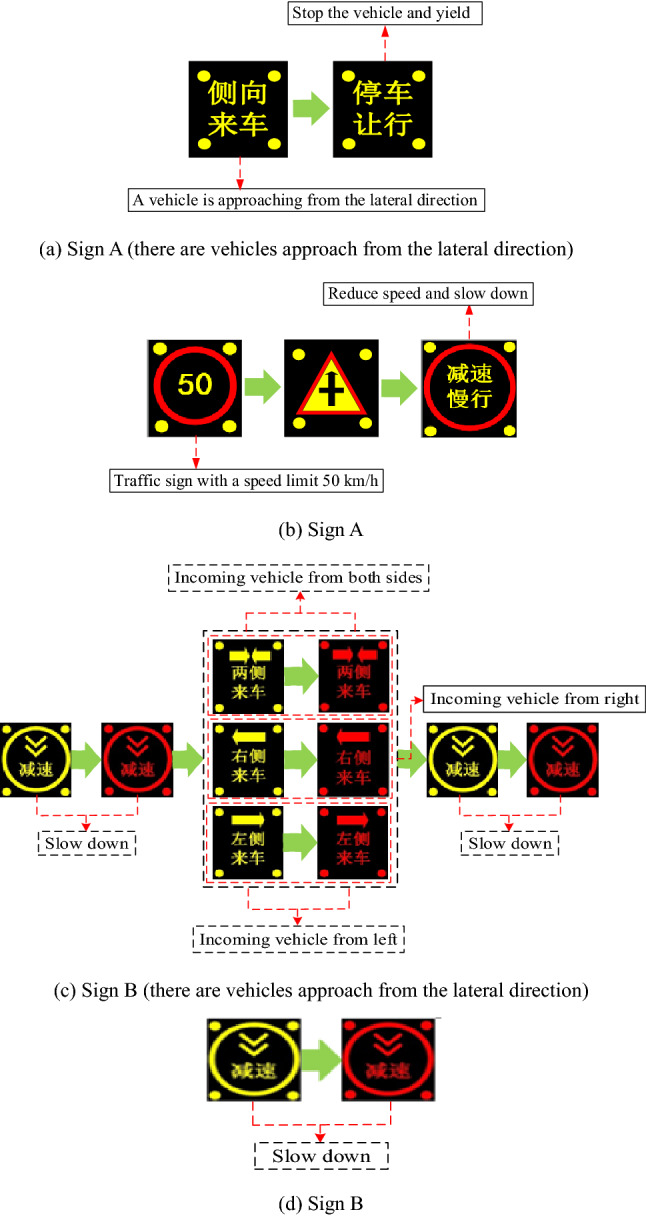
Two types of warning signs.

Sign A is shown in Fig. 6a,b. When there are vehicles approaching from the lateral direction, the information graph (Fig. 6a) is flashed on the warning sign in a sequence, with a flashing frequency of 0.16 s and a period of 1 s. When there is no conflict vehicle in the lateral direction, the information graph (Fig. 6b) is flashed on the warning signs in a sequence, with a flashing frequency of 0.16 s and a period of 1 s.

Sign B is shown in Fig. 6c,d. When there are vehicles approaching from the lateral direction, three strategies are employed for the vehicle on both sides, the vehicle on the right, and the vehicle on the left. The information graph (Fig. 6c) is flashed on the warning sign in a sequence, with a flashing frequency of 0.16 s, and a period of 1 s. When there is no conflict vehicle from the lateral direction, only the information graph (Fig. 6d) is flashed, with a flashing frequency of 0.16 s, and a period of 1 s.

### Procedures

**Figure 7:**
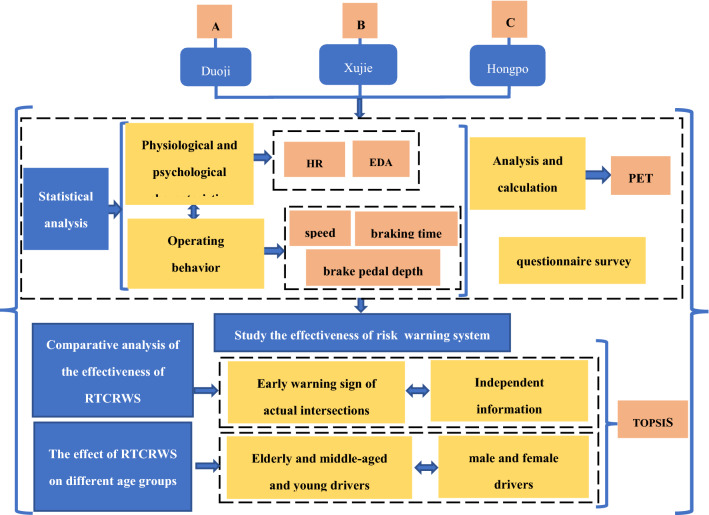
Research methods and steps.

The methods and indicators used, as well as the main research ideas, are shown in Fig. 7. In this study, driving simulation experiments were adopted. Before the start of the experiment, it was necessary to ensure that the driving simulation system was in good working condition and that the physiological and psychological instrument signals were being received normally. Simultaneously, ensuring the synchronisation of each piece of experimental equipment in time was necessary to obtain and record synchronous physiological, psychological, and driving data. Afterwards, the participants needed to understand the goal of the experiment and to be familiar with the use of the driving simulator. Furthermore, the experiment guides briefly introduced the function of the warning system to the test drivers, and the drivers were advised to complete the driving simulation experiment according to their own driving habits and response characteristics. All methods were carried out in accordance with relevant guidelines and regulations and all experimental protocols were approved by the ethics committee of National science and technology ethics committee of China and Discipline Inspection and Supervision Department of Yunnan Transportation Planning and Design Research Institute Co., Ltd.

### Indicator selection and data pre-processing

Speed is an intuitive expression of driving behavior in vehicle operation, and an important indicator that affects driving safety and comfort. When encountering a risk, drivers who drive too fast will adopt emergency braking or steering behaviors, thus reducing the safety and comfort of driving, and increasing the possibility of accidents. When the speed is too low, although safety can be guaranteed to a certain extent, the time to complete the driving task will be greatly increased, and its comfort will be reduced. In addition, when drivers encounter risks in the process of completing driving tasks, they generally adopt braking measures to avoid collisions. The braking behavior of drivers is mainly represented by the brake pedal depth, and the standard deviation of the brake pedal depth can reflect the driver's ability to control vehicle deceleration. When the driver perceived the risk earlier, the brake force was smaller, the brake pedal depth was shallower, and the braking distance was longer. When the driver perceives the risk later, the driver braking force is larger, the brake pedal depth is deeper, and the braking distance is shorter. And in the process of driving, drivers are affected by traffic flow, driving time, road environmental conditions, vehicle conditions and other factors, their physiology and psychology will change. Physiological and psychological changes can reflect the state of the driver in the process of driving tasks. The larger the change, the greater the driver's emotional fluctuations, and the smaller the change, the more stable the driver's driving state.

In this study, speed, maximum brake pedal depth and earliest brake time are selected as indicators to characterize vehicle operation, while HR and EDA are selected as indicators to characterize drivers' physiological and psychological characteristics. Considering the design of no steering on the driving road in the experimental scene and the setting of the risk warning system at the intersection, the lateral safety indexes are not extracted.

During the driving simulation experiment, the vehicle operation and physiological and psychological data were obtained through the data acquisition system of each device. A total of 38 drivers participated in this experiment, and the driving simulation experiments were conducted while the drivers were wearing a physiological psychology instrument.

Through the driving simulation platform and Ergo LAB, vehicle operation and physiological and psychological data were extracted and analysed. After preliminary data sorting, a total of 4644 EXCEL data sheets were created for the 38 drivers’ driving operations, and physiological and psychological data files.

### Validation of the experimental platform

**Figure 8:**
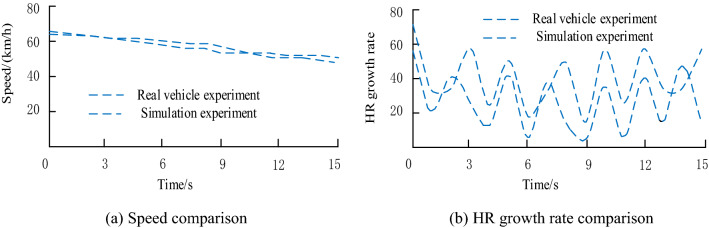
Verification of the effectiveness of the experimental platform.

Vehicle speed and driver's physiological and psychological reaction^22,23^ are important indicators to characterize the effectiveness of driving simulation experiments. In this study, the effectiveness of driving simulation experiment was verified by comparing the vehicle speed and the driver's heart rate growth rate in real and virtual scenarios. Vs-design scene Design software was used to deeply restore the actual intersection situation, and intersection A was selected for real vehicle test. The comparison results of the two groups of experiments are shown in Fig. 8.

It can be seen from Fig. 8a,b that the variation trend of average speed in the simulation experiment is consistent with that in the real vehicle experiment, which is slightly higher than that in the real vehicle experiment overall. In addition, the variation trend of driver's heart rate growth rate in the simulation experiment and the real vehicle experiment is similar. The changes of the two indexes are consistent, indicating that the driving simulation system has a relatively high effectiveness in the implementation of driving behavior related research.

### Ethical approval

The data used in this study is provided by the Simulation Laboratory of Faculty of Transportation in Kunming University of Science and Technology, Kunming, Yunnan Province, China.

## Results

### Driving data analysis

#### Speed

The speed changes and driving status of each subject at intersections A, B, and C are statistically analyzed. Determine whether the driver is in a safer driving state by analyzing whether the driver slows down to pass the intersection. The driver who decelerates through the intersection is in a safe driving state, and the driver who keeps driving at high speed when passing through the intersection is judged to be in a dangerous driving state.

**Figure 9:**
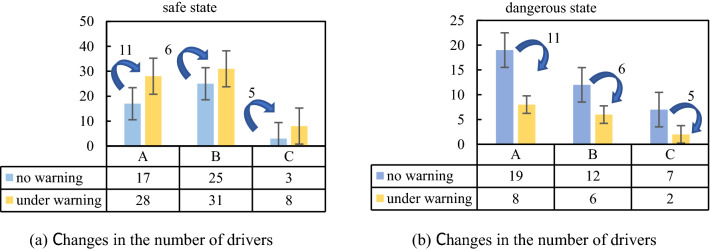
Distribution of the number of people in safe and dangerous state at 3 intersections.

At intersection A, there were 36 effective drivers. When there was a risk warning system, the number of drivers in the safe driving state increased by 65%, and the number of drivers in the dangerous driving state decreased by 58%; at intersection B, the number of effective drivers was 37. When there was a risk warning system, the number of drivers in the safe driving condition increased by 24%, and the number of drivers in the dangerous driving state decreased by 50%; at intersection C, the number of effective drivers was 10. With the risk warning system, the number of drivers in the safe driving state increased by 63%, and those in the dangerous driving state driver decreased by 71%. The results are shown in Fig. 9.

#### Brake pedal depth

**Figure 10:**
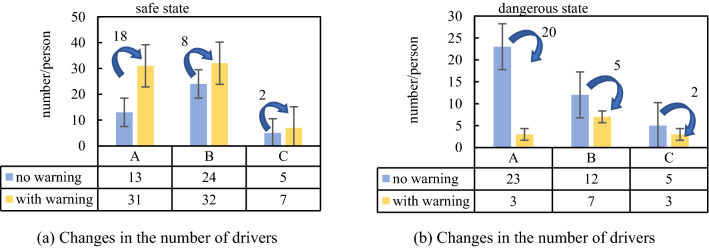
Distribution of safer and dangerous drivers at 3 intersections with or without warning.

The depth of the brake pedal can reflect whether a driver brakes slowly in advance or brakes urgently. If a driver brakes slowly in advance, it indicates that the driver has foreseen a risk and has reacted in advance, while emergency braking indicates that the driver did not foresee the risk in advance and therefore took emergency measures. According to the braking reaction time data of 38 drivers collected in this experiment, and combined with the time when the test driver observed the sign, we divided drivers into two types: drivers with a reaction time of “0–20 s” were considered safe drivers and those with a reaction time “over 20 s” were considered dangerous drivers, the types of drivers at the three intersections were analysed. At intersection A, when there was a risk warning system, the number of safe drivers increased by 58%, while that of dangerous drivers decreased by 87%; at intersection B, when there was a risk warning system, the number of safe drivers increased by 33%, while the number of dangerous drivers decreased by 42%; at intersection C, when there was a risk warning system, the number of safe drivers increased by 40%, while that of dangerous drivers decreased by 40%. The results are presented in Fig. 10.

#### Earliest braking time

Brake reaction time refers to the time from the driver's perception to the risk of a conflict to taking braking measures. The earliest braking time refers to the earliest time the driver begins to take braking measures after predicting the risk of conflict, and can reflect the driver's response speed to an emergency to a certain extent. As shown in Table 1, the earliest braking times at the three intersections were evidently advanced. After setting up the risk warning system, the earliest braking times at intersections A, B, and C were advanced by 7.175, 2.329, and 3.313 s, respectively.

**Table 1:** Comparison of the earliest braking times.

	With/without RTCRWS	A	B	C
Earliest braking time (s)	No warning	15.673	13.801	14.411
	With warning	8.498	11.472	11.098
	Sig	0.000**	0.185	0.501

*Sig*. significance level of T-test.

*Significant at 0.05 level.

**Significant at 0.01 level.

#### Post-encroachment time

Post-encroachment time (PET) refers to the difference between the time when the rear vehicle enters the potential conflict area and the time the vehicle in front leaves the area. It is often used to measure the traffic conflict at the point where driving tracks intersect. The calculation is shown in Eq. (2). A PET value of 0 indicates that a collision has occurred; the closer the PET value is to 0, the higher the risk of conflict.

2 $$PET={t}_{B}-{t}_{A}$$

${t}_{B}$—The moment when the vehicle with the right of way arrives at the point of conflict.

${t}_{A}$—The moment when the offending vehicle passes through the point of conflict.

**Figure 11:**
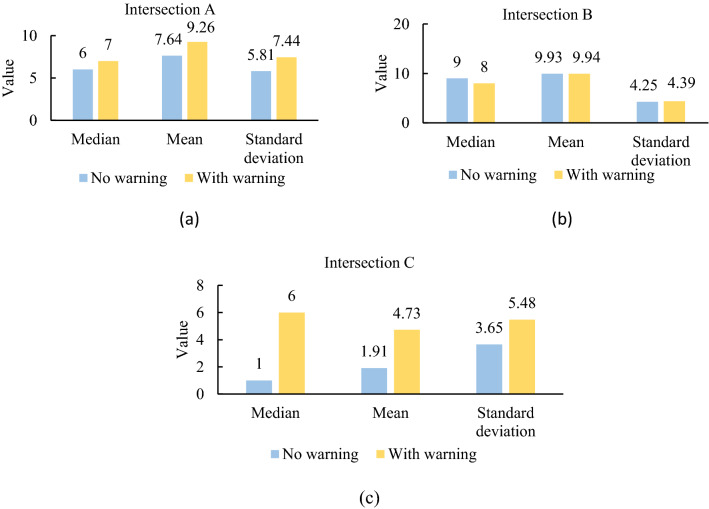
PET values of 3 intersection.

According to the video recorded in the experiment, the effective data points that can be used to calculate PET are as follows: at intersection A, for the scenario with and without a risk warning systems, the data points were 28 and 43, respectively; at intersection B, for the scenario with and without a risk warning systems, the data points were 28 and 43, respectively; and at intersection C, for the scenario with and without a risk warning systems, the data points were 11 and 11, respectively. The PET of each intersection with or without a risk warning system was analysed for statistical differences, and the median, mean, and standard deviation of the PET were calculated (Fig. 11). After setting up the risk warning system, the mean and median of the PET at intersections A, B and C increased, indicating that the warning system can enable drivers to perceive risks in advance and take appropriate measures. When there was a risk warning system, the PET standard deviation of the three intersections increased, which to a certain extent indicates that the presence of a risk warning system at each intersection made the PET change significantly.

### Physiological and psychological data analysis

#### Heart rate (HR)

Heart rate (HR) refers to the number of heart beats per minute in normal people's quiet state and is usually 60–100 beats per minute. Due to individual differences in age, gender, or other physiological factors, the HR of the drivers in the driving processes can directly reflect their psychological tension. As shown in Table 2, after setting up the risk warning system, at intersections A and B, the average HR of the driver increased, while the HR growth rate decreased, the average HR decreased, and the HR growth rate increased. An independent sample t-test revealed that there was no significant difference in the average HR and HR growth rate of the drivers between the two scenarios.

**Table 2:** Comparison of HR and growth rate.

Intersection name	With/without RTCRWS	Mean (Bpm)	Growth rate
A	No warning	74.63	0.03
	With warning	76.11	0.026
	Sig	0.565	0.811
B	No warning	70.70	0.089
	With warning	75.52	0.020
	Sig	0.085	0.078
C	No warning	71.17	0.0243
	With warning	73.26	0.0176
	Sig	0.671	0.486

*Sig*. significance level of T-test.

#### Electrodermal activity (EDA)

Electrodermal activity (EDA) is an indirect index that reflects the degree of sympathetic nerve activity and can be used to evaluate the emotional arousal level and psychological activity of drivers. A positive correlation exists between EDA and the degree of the physical tension of drivers; the more nervous a driver is, the higher is their EDA, and the more relaxed the driver is, the lower is their EDA. After setting up the risk warning system, the mean value of the EDA of the drivers decreased at intersections A, B, and C. However, the independent sample t-tests, as summarised in Table 3, revealed that there were no significant differences in the mean, median, maximum, and minimum values of the EDA.

**Table 3:** Comparison of the characteristic values of skin electricity.

Intersection name	With/without RTCRWS	Mean	Median	Max	Min
A	No warning	2.82	2.10	14.06	0.38
	With warning	2.62	1.71	11.56	0.40
	Sig	0.664	0.716	0.619	0.568
B	No warning	5.10	5.06	5.63	4.50
	With warning	5.08	5.06	5.84	4.60
	Sig	0.991	1.000	0.900	0.925
C	No warning	5.89	5.90	12.86	5.28
	With warning	5.36	5.30	6.64	5.31
	Sig	0.683	0.648	0.896	0.861

*Sig*. significance level of T-test.

### Subjective questionnaire

**Figure 12:**
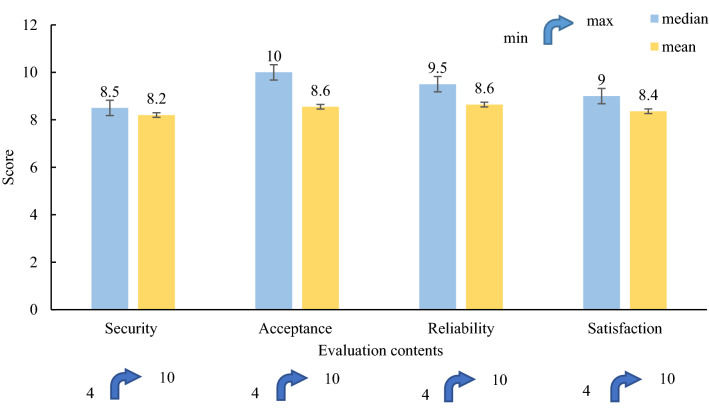
Survey scores for the effectiveness of the RTCRWS.

Through a questionnaire survey, the drivers’ basic information and the drivers’ level of recognition and acceptance of the RTCRWS were determined. These data were used to further evaluate the effectiveness of the system. The ages of the driver varied from 21–72 years old, with an average age of 50 years. The driving experience of the drivers was between 2 and 44 years, with an average of 19 years. The driving mileage was between 20,000 km and 2 million kilometres, and the average was 280,000 km. Before and after the driving simulation experiment, the basic information of drivers and the early warning system evaluation questionnaire were conducted respectively. The statistical results of the evaluation scores the drivers gave the warning system are shown in Fig. 12, including four evaluation contents: safety, acceptance, reliability, and satisfaction of the early warning system. According to the results of the effectiveness survey of the warning system, most drivers gave the early risk warning signs a high evaluation, and the average scores of the four items were 8.2, 8.55, 8.64, and 8.36, respectively, reflecting the driver's subjective attitude toward the early risk warning signs.

### Effectiveness evaluation

#### Topsis algorithm

Effectiveness was evaluated using the technique for order preference similarity to ideal solution (Topsis) algorithm. Topsis is a commonly used method for multi-objective decision analysis of limited schemes that can be used in the fields of benefit evaluation and decision management. It is based on the normalised original data matrix, and the cosine method is used to determine the optimal scheme and the worst scheme in the limited scheme (expressed by the optimal vector and the worst vector, respectively). Then, the distance between each evaluation object and the optimal scheme and the worst scheme is calculated, and the relative proximity between each evaluation object and the optimal scheme is obtained, which is used as the basis for evaluating the quality.

#### Algorithm steps

1. Process the index attributes to ensure data convergence.
  Convert all low-quality indicators and neutral indicators into high-quality indicators ${x}_{ij}$, and appropriately adjust them (increase or reduce them based on a certain ratio) to converge the data.
  $${x}_{ij}=\left\{\begin{array}{l}{\mathrm{x}}_{\mathrm{ij}} High-quality\,index\\ \frac{1}{{\mathrm{x}}_{\mathrm{ij}}} Low-quality\,index\\ \frac{\mathrm{M}}{\mathrm{M}+|{\mathrm{x}}_{\mathrm{ij}}-\mathrm{M}|} Neutral \,index\end{array}\right.$$
2. Normalise the processed data.
  $${Z}_{ij}=\left\{\begin{array}{l}\frac{{\mathrm{x}}_{\mathrm{ij}}}{\sqrt{\sum_{\mathrm{i}=1}^{\mathrm{n}}{({\mathrm{x}}_{\mathrm{ij}})}^{2}}} High\,quality\,index \\ \frac{{\mathrm{x}}_{\mathrm{ij}}}{\sqrt{\sum_{\mathrm{i}=1}^{\mathrm{n}}{({\mathrm{x}}_{\mathrm{ij}})}^{2}}} low\,quality\,or\,neutral\,index\end{array}\right.$$
3. Determine the best and worst plans.
  The optimal solution ${Z}^{+}$ entails the maximum value in each column of Z:${Z}^{+}=\left(max{Z}_{i1},max{Z}_{i2},\dots ,max{Z}_{im}\right)$, where ${Z}^{-}$ is the smallest value in each column of Z:${Z}^{-}=(min{Z}_{i1},min{Z}_{i2},\dots ,min{Z}_{i3})$.
4. Calculate the distance ${D}_{i}^{+}$ and ${D}_{i}^{-}$ between each evaluation object and ${Z}^{+}$ and ${Z}^{-}$.
  $${\mathrm{D}}_{\mathrm{i}}^{+}=\sqrt{\sum_{\mathrm{i}=1}^{\mathrm{m}}{({\mathrm{maxZ}}_{\mathrm{ij}}-{\mathrm{Z}}_{\mathrm{ij}})}^{2}}$$
  $${\mathrm{D}}_{\mathrm{i}}^{-}=\sqrt{\sum_{\mathrm{i}=1}^{\mathrm{m}}{({\mathrm{minZ}}_{\mathrm{ij}}-{\mathrm{Z}}_{\mathrm{ij}})}^{2}}$$
5. Calculate the closeness ${\mathrm{C}}_{\mathrm{i}}$ of each evaluation object to the optimal solution.
  $${\mathrm{C}}_{\mathrm{i}}=\frac{{\mathrm{D}}_{\mathrm{i}}^{-}}{{\mathrm{D}}_{\mathrm{i}}^{+}+{\mathrm{D}}_{\mathrm{i}}^{-}} 0\le {\mathrm{C}}_{\mathrm{i}}\le 1$$
  ${\mathrm{C}}_{\mathrm{i}}$→1 shows that the evaluation object is better.
6. Sort by ${\mathrm{C}}_{\mathrm{i}}$ size and provide the evaluation results.

#### Comparison and analysis of the evaluation results

The HR, EDA, speed, maximum brake pedal depth, and earliest braking time were selected as the evaluation indexes, and the Topsis method was used to evaluate the effectiveness of RTCRWS.

The effectiveness of the actual intersection early warning signs and independent information combined with early warning signs were compared and evaluated. The results are shown in Table 4. According to the evaluation and ranking results of the Topsis method, compared with that of the actual intersection early warning signs, the warning effect of independent information combined with the early warning signs was better.

**Table 4:** Effectiveness evaluation results for two types of early warning signs.

	$\mathrm{D}+$	$\mathrm{D}-$	${\mathrm{C}}_{\mathrm{i}}$	Sort results
Sign A	0.1378	0.0223	0.1392	2
Sign B	0.0223	0.1378	0.8608	1

**Figure 13:**
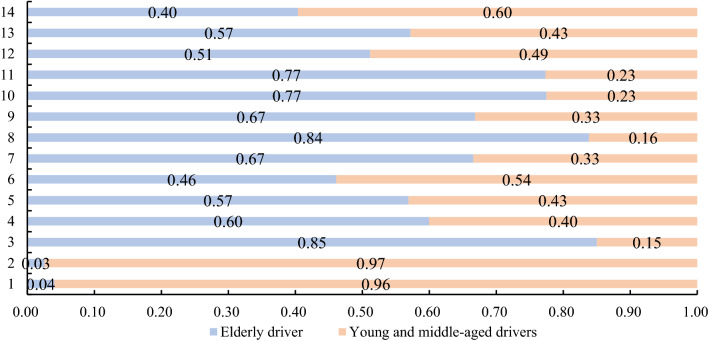
Effect score of the RTCRWS for elderly and middle-aged and young drivers.

**Figure 14:**
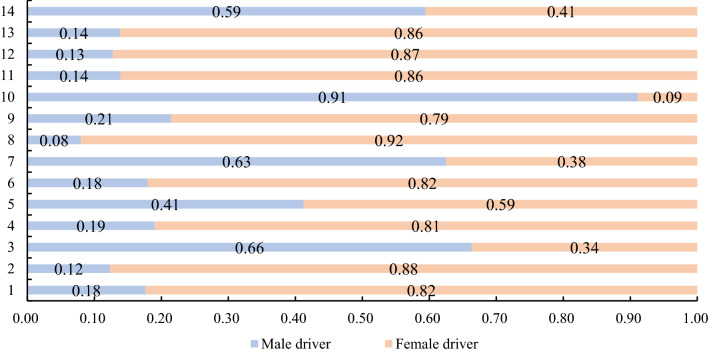
Effect score of the RTCRWS for male and female drivers.

The warning effect of the RTCRWS was compared and evaluated for the elderly and middle-aged and young drivers, and the scoring results are shown in Fig. 13. Finally, a comparative evaluation of the effectiveness of the risk warning system on the warning effect of male and female drivers was carried out, and the scoring results are shown in Fig. 14. By analysing the evaluation results of intersections A and B, and the 12 intersections on simulated experimental roads (Table 5), it was found that RTCRWS had a better warning effect on elderly drivers than on middle-aged and young drivers; furthermore, the warning effect of the RTCRWS on female drivers was better than that on male drivers.

**Table 5:** Valuation results of the warning effect of the RTCRWS on different drivers.

	1	2	3	4	5	6	7	8	9	10	11	12	13	14	Total
Elderly driver			√	√	√		√	√	√	√	√	√	√		10
Young and middle-aged drivers	√	√				√								√	4
Male driver			√				√			√				√	4
Female driver	√	√		√	√	√		√	√		√	√	√		10

## Discussion

In this study, the effectiveness of RTCRWS for the intersections on mountain roads that are not signal controlled was evaluated. First, driving simulation experiments and questionnaire surveys were conducted, and physiological and psychological parameters and vehicle operating parameters were selected as research indicators to analyse the effectiveness of the system.

First, the driving data (e.g., speed, brake pedal depth, earliest braking time) were analysed. According to the previous conclusion, after RTCRWS was established, safe drivers significantly increased, while dangerous drivers significantly decreased. As shown in Fig. 10, drivers can anticipate risks and start slowly braking in advance rather than taking emergency braking measures. The system had a good risk prediction ability and deceleration effect at the intersections that are not signal controlled, and the risk of road conflict decreased after RTCRWS was set up, as shown by the calculated PET (Fig. 11), indicating that the risk warning system can effectively improve driving safety.

The HR and EDA indices of the driver were then analysed. The results revealed that the mean value of the HR increased and the mean value of EDA decreased when the driver observed the risk early warning sign, as shown in Tables 2 and 3. These results indicate that RTCRWS had warning effect on the driver, as it enabled the driver to know the state of the intersection in advance. Corresponding braking deceleration measures can be taken by drivers to pass through the intersection in a stable and safe state, indicating that the risk warning system can improve the physiological and psychological adaptation abilities of drivers to a certain extent. Through independent sample T-test, however, it was found that there were no significant differences in the mean, median, maximum, and minimum values of EDA, indicating that the warning effect of RTCRWS on drivers cannot be effectively judged by the EDA index alone.

Finally, the HR, EDA, speed, maximum brake pedal depth, and earliest braking time were selected as evaluation indexes, and the Topsis method was used to evaluate the effectiveness of RTCRWS. The effect of the sign B was better than sign A, as shown in Table 4, the display strategy of this type signs can provide some reference for the information design of early warning signs. The effectiveness of RTCRWS was evaluated for elderly and middle-aged and young drivers, and the results revealed that the warning effect for the elderly was better (Fig. 13), while for male and female drivers, the results revealed that the warning effect of female drivers was better (Fig. 14).

This study also had limitations, one of which was that the differences in the behaviour characteristics of drivers classified according to the depth of brake pedals were not analysed. Future studies will explore the cognitive responses of different types of drivers to RTCRWS, including drivers with different driving styles and habits. The physiological, psychological, and driving data in this study were collected in the driving simulation experiment based on virtual scenarios. As shown in Fig. 12, most drivers recognised the effectiveness of the risk warning system, reflecting the driver's subjective attitude toward RTCRWS. However, there are some differences between virtual and actual roads. A real car experiment will be carried out in a future study, and the data from the simulation experiment will be compared with the experimental data. Simulation experiments and real vehicle experiments will be combined to verify the effectiveness of RTCRWS. In addition, only the HR and EDA were considered in the selection of physiological and psychological indicators. In a future study, other physiological and psychological indicators, and driver's eye movement data will be considered.

## Conclusions

In this study, a driving simulation experiment was performed to collect drivers' driving and physiological and psychological data of drivers. Together with questionnaire survey data on the effectiveness of warning system and warning signs, it was used to determine the effectiveness of RTCRWS. At the same time, the two types of early warning signs of RTCRWS were compared and analyzed. The conclusions of this study are as follows:

1. After RTCRWS was set up, the physiological and psychological state of the drivers tended to stabilise, and braking measures were taken in advance to control the vehicle speed within a controllable range. The proposed risk warning system had good risk prediction ability and could effectively remind the driver to decelerate to ensure driving safety. Moreover, to a certain extent, it could improve the physiological and psychological adaptability of driving.
2. In this study, two types of early warning signs were put forward, and the Topsis method was used to compare and analyse effectiveness. The warning effect of the sign B was better than sign A.
3. In this study, the proportion of elderly drivers and middle-aged and young drivers recruited in the driving simulation experiment was 8:11. For drivers of these two age groups, the effectiveness of RTCRWS was studied based on physiological and psychological characteristics and driving behaviour. The results revealed that RTCRWS was more effective for elderly drivers.
4. For male and female drivers, HR, EDA, speed, maximum brake pedal depth, and earliest braking time were used as evaluation indicators, and the data from 14 intersections was the research basis. The analysis results of the Topsis method revealed that RTCRWS had a better warning effect for female drivers.

## Data Availability

The datasets generated and/or analysed during the current study are not publicly available due data use requires permission from experimental collaborators but are available from the corresponding author on reasonable request.
